# PTEN-mediated AKT/β-catenin signaling enhances the proliferation and expansion of Lgr5+ hepatocytes

**DOI:** 10.7150/ijbs.56091

**Published:** 2021-02-17

**Authors:** Jimin Han, Kaijun Lin, Xuezheng Zhang, Lingchen Yan, Jianjun Liu, Jia Liu

**Affiliations:** 1 School of Pharmaceutical Sciences (Shenzhen), Sun Yat-sen University, Guangzhou, China.; 2 Medical Key Laboratory of Health Toxicology of Shenzhen, Shenzhen Center for Disease Control and Prevention, 518054, Shenzhen, China.

**Keywords:** AKT/β-catenin, Lgr5, hepatocyte, proliferation, liver regeneration.

## Abstract

**Rationale**: Compelling evidence suggests that Lgr5+ hepatocytes repair liver damage by promoting the regeneration of hepatocytes and ductal cells in the case of liver injury. The PTEN-mediated AKT/β-catenin signaling plays a key role in the regulation of innate immune regulation in the liver. However, the signaling pathways that control Lgr5+ hepatocyte proliferation in the liver remain unclear.

**Methods**: In order to assess the involvement of PTEN-mediated AKT/β-catenin signaling in the expansion of Lgr5+ hepatocytes upon liver injuries, the Lgr5-CreER; Rosa-mTmG lineage tracing system was used to target Lgr5+ hepatocytes.

**Results:** The tracing of Lgr5+ hepatocytes showed that PTEN deletion and β-catenin activation significantly promoted the proliferation of Lgr5+ hepatocytes. In converse, the simultaneous inhibition of PTEN and β-catenin limited Lgr5+ hepatocyte proliferation in the liver. Our findings provide an insight into understanding how PTEN-mediated AKT/β-catenin signaling regulates the proliferation of Lgr5+ hepatocytes.

**Conclusion:** The outcomes can improve the application potential of Lgr5+ hepatocytes in the treatment of liver injury diseases and provide a new treatment option for liver cancer.

## Introduction

The target gene of Wnt signaling, Lgr5, has been shown to be a tissue stem cell marker for maintaining organ homeostasis, including the small intestine 1-8, colon 9-15, antral stomach 15-18, hair follicles 19, 20, mammary gland 21, 22, and ovary 23. Using canonical lineage analysis system and bacterial artificial chromosome (BAC)- transgenic model with a high labeling efficiency, Lgr5 has also been documented on a unique subset of damaged induced hepatocytes 24, 25. In the liver, Lgr5+ hepatocytes are an important cell population that contributes to the maintenance of liver homeostasis and damage repair and are mainly distributed around the central vein 24, 26. Lgr5+ hepatocytes are long-lived, maintaining their own lineage and long-term presence in homeostasis and liver injury 24. When the liver suffers an injury by CCl_4_, 3,5-diethoxycarbonyl-1,4-dihydrocollidine (DDC), and other toxic substances, Lgr5+ hepatocytes that possess the potential of bidirectional differentiation can differentiate into hepatocytes and bile duct cells 25. At present, *in vitro* culture systems that generate liver organoid from isolated mouse Lgr5+ hepatocytes have been established 25, and long-term culture and large-scale amplification of liver organoids have also been achieved 27.

The exact mechanism by which damage induces Lgr5+ hepatocyte proliferation remains a mystery. Since Lgr5 plays an important role in enhancing the activity of Wnt/β-catenin signaling pathway, high-intensity Wnt signaling after a liver injury is likely to indicate Lgr5+ hepatocyte proliferation to repair a damaged liver. PTEN negatively regulates the PI3K-AKT signaling pathway, an essential pathway for the regulation of cell survival and proliferation 28, 29, which consequently leads to nuclear translocation of β-catenin through phosphorylation of GSK-3β 30. PTEN-mediated AKT/β-catenin signaling controls inflammatory damage in mouse liver triggered by ischemia/reperfusion injury and regulates the release of inflammatory cytokines in the liver 31. Meanwhile, PTEN regulates NK cells and Kupffer cells in the liver to modulate the proliferation of hepatocytes to promote liver repair 32. However, the role of PTEN in Lgr5+ hepatocytes mediated liver regeneration remains to be explored.

A better understanding of the molecular mechanisms by which Lgr5+ hepatocytes promote liver regeneration is critical. In this study, Lgr5+ hepatocytes in the liver were tracked using the Lgr5-CreER; Rosa-mTmG (referred to as Lgr5CreER; mTmG) inducible lineage analysis system under the conditions of PTEN inhibition and β-catenin overexpression. We found that both PTEN deletion and β-catenin overexpression induced the proliferation of Lgr5+ hepatocytes, indicating the pivotal role of PI3K/AKT and Wnt/β-catenin signaling pathways. We also observed that β-catenin inhibition significantly reduced the effects of PTEN signaling activation in Lgr5+ hepatocytes, suggesting that PTEN-mediated PI3K/AKT signaling synergizes with β-catenin signaling to enhance Lgr5+ hepatocyte expansion. These findings suggested that PTEN-mediated AKT/β-catenin signaling may act as a signal released by liver injury, and Lgr5+ hepatocytes proliferate and differentiate into liver cell lineages to repair the liver under stimulation of this signal. This study may provide a potential target for promoting Lgr5+ hepatocyte proliferation to repair the damaged liver.

## Results

**Figure 1:**
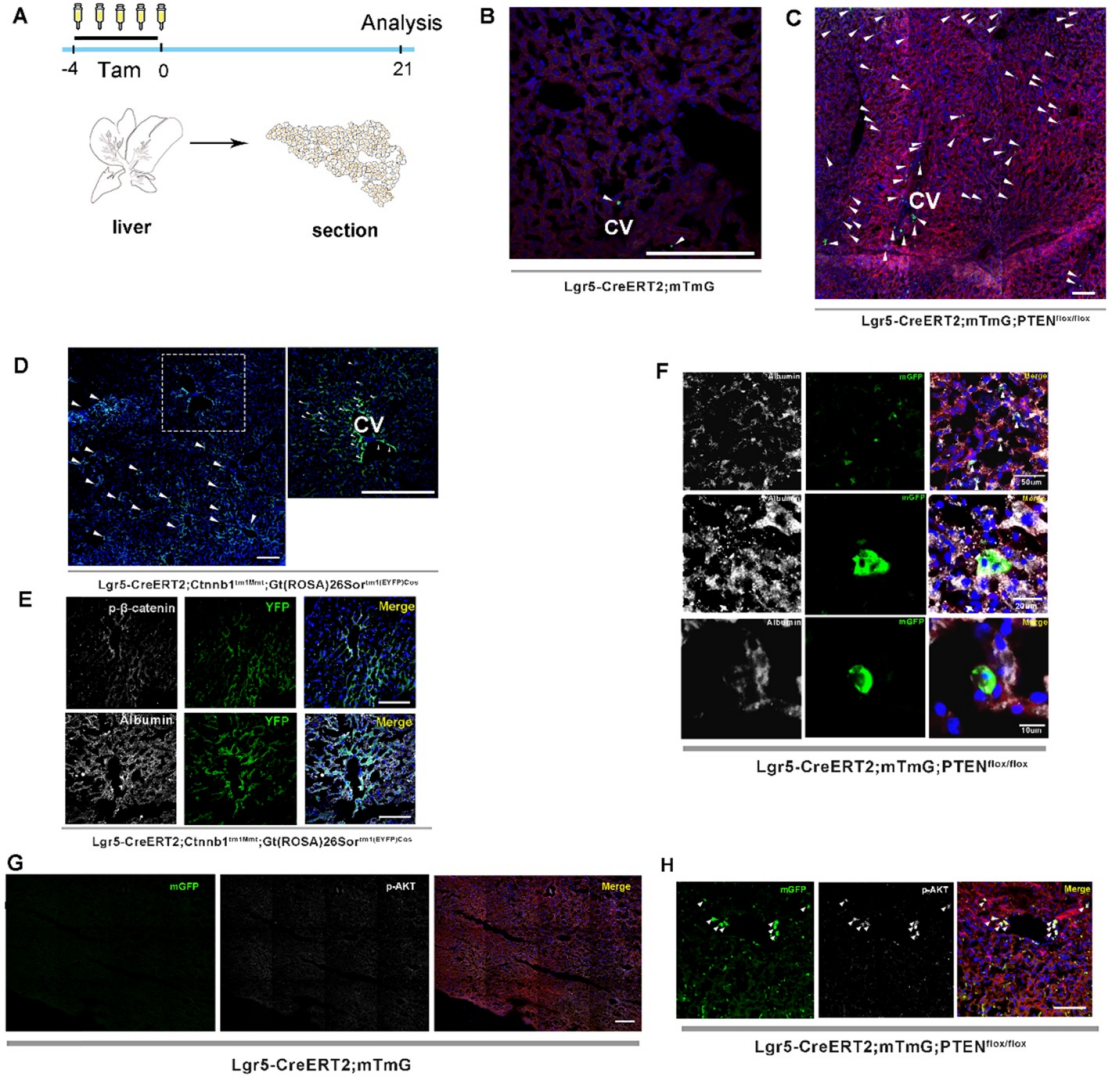
** Both PTEN ablation and increased β-catenin signaling in Lgr5+ hepatocytes promoted the expansion of Lgr5+ hepatocytes.** (A) Experimental scheme for tamoxifen injection and analysis of liver samples in Lgr5CreER; mTmG mice at 21 days post-injection. (B) Targeting of Lgr5+ hepatocytes and their progeny (green) in the liver with Lgr5CreER; mTmG reporter mice. Note the sporadic distribution of mGFP-labeled cells in the liver. (C) Representative images showing positive Lgr5+ hepatocytes and their progeny in Lgr5CreER; mTmG; PTEN^flox/flox^ mice. (D) A low-magnification image of a liver sample from Lgr5CreER; Ctnnb1^Δ(ex3)^; YFP mice (left panel). A magnified image of the left panel showed labeled Lgr5+ hepatocytes (right panel). Arrowheads indicate mGFP+ hepatocytes on day 21 after injection. (E) Phosphorylated β-catenin (white) immunostaining of liver tissue showing the specific colocalization of p-β-catenin and YFP (upper panel). Immunostaining of liver section from Lgr5CreER; Ctnnb1^Δ(ex3)^; YFP mice showed that mGFP-labeled cells colocalized with Albumin (white) (lower panel). (F) Immunostaining for Albumin (white) revealed colocalization of mGFP with Albumin-positive hepatocytes. (G-H) Immunostaining of liver tissues from Lgr5CreER; mTmG and Lgr5CreER; mTmG; PTEN^flox/flox^ mice for p-AKT antibody (white). Arrowheads indicate Lgr5+ hepatocytes with an activated AKT pathway. n = 3 mice for each genotype; CV: central vein; Scale bar: 50 µm.

**Figure 2:**
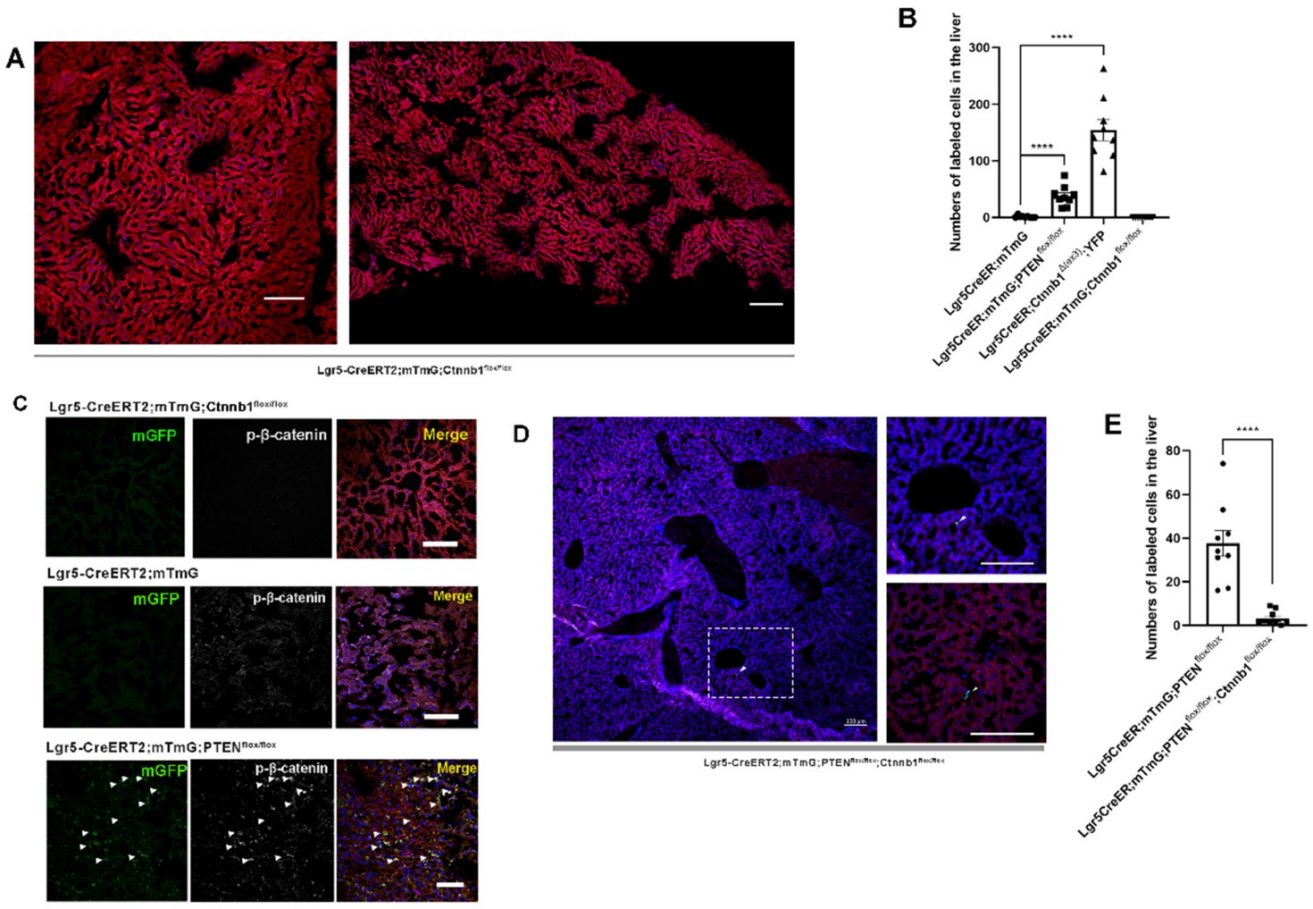
** PTEN-mediated AKT/β-catenin signaling controls Lgr5+ hepatocyte proliferation.** (A) The number of labeled cells in the liver from Lgr5CreER; mTmG; Ctnnb1^flox/flox^ mice was significantly reduced upon β-catenin deletion. (B) Quantitation of mGFP+/YFP+ cells in liver samples from mice with different genotypes. (C) p-β-catenin immunostaining to show activation or inhibition of β-catenin signaling in the liver from different genotypes. (D) Sections of liver in Lgr5CreER; mTmG; PTEN^flox/flox^; Ctnnb1^flox/flox^ mice showing that β-catenin deletion in PTEN-deficient Lgr5+ hepatocytes strongly blocked the proliferation of Lgr5+ hepatocytes (left panel). The high-magnification view of the left panel depicts sporadic mGFP+ cells (top right). Another higher magnification image showing Lgr5-labeled cells (bottom right). Arrowheads denote mGFP+ cells. (E) Quantitation of mGFP+ cells in liver samples from mice with different genotypes. ****, P<0.0001. Data shown are means ± SEM (n = 3 biological replicated experiments). Scale bar: 100 µm.

Previous studies on lineage tracing of Lgr5+ hepatocytes for 18 months revealed that only a few cloned cells (1-5 cells) were produced by a single Lgr5+ hepatocyte, indicating a low level of Lgr5+ cell division during homeostasis 24, 26. Moreover, the lineage tracing of Lgr5+ hepatocytes using traditional LGR5-IRES-creERT2 reporter mice showed that Lgr5 expression was not detected in healthy liver, but only in bile duct after injury 25, suggesting that Lgr5+ hepatocyte proliferation may be a stress response to the liver repair. In this study, we attempted to determine whether altering PTEN-mediated AKT signaling in Lgr5+ hepatocytes would affect Lgr5+ cell proliferation. Lgr5CreER; mTmG mice were crossed with PTEN^flox/flox^ mice to generate Lgr5CreER; mTmG; PTEN^flox/flox^ mice (Figure S1). Tamoxifen was injected for five consecutive days to specifically knock out PTEN in Lgr5+ hepatocytes to activate AKT signaling and simultaneously trace Lgr5+ hepatocytes (Figure 1A). Tamoxifen administration resulted in permanent membrane GFP expression (mGFP+) within Lgr5-expressing cells. Twenty-one days after injection, we were surprised to find an increased distribution of mGFP-labeled cells in the liver (Figure 1C and 2B, Figure S2A-E) and that these mGFP-labeled cells colocalized with Albumin (a marker of hepatocytes) (Figure 1F). Immunostaining for phosphorylated AKT (p-AKT) also showed significant AKT signaling activation in mGFP-labeled cells (Figure 1H). While in Lgr5CreER; mTmG control mice, we only found sporadic mGFP-labeled cells (Figure 1B and 2B, Figure S2F-H). These data suggested that the activated AKT signaling promoted the expansion of Lgr5+ hepatocytes. To test the efficacy of tamoxifen, we also observed the tracking of Lgr5+ cells in the colon, intestine, and hair follicles. After five days of injection, hair follicle stem cells expressing Lgr5, as well as stem cells in the colon and intestinal crypts and their progenies, were all labeled with mGFP and showed high labeling efficiency (Figure S4A-E). As previously reported 24, the traditional tracing system of Lgr5CreER; mTmG mice could not obtain the high tracing efficiency of the novel BAC transgenic mice since the traditional tracing system cannot mimic the true expression pattern of Lgr5 at mRNA levels in the liver 25.

Upon hepatectomy or central venous injury, Wnt signaling in the liver is highly activated 25, 33. An injury to the liver is often accompanied by Lgr5+ hepatocyte expansion and high Wnt signaling activity 25. Moreover, Lgr5 is not only a target gene of Wnt/β-catenin signaling but also regulated by this pathway to maintain stemness. Therefore, we suspected that β-catenin plays a key role in regulating the proliferation of Lgr5+ hepatocytes. Next, we crossed Lgr5CreER and Ctnnb1^tm1Mmt^; Gt(ROSA)26Sor^tm1(EYFP)Cos^ mice to generate conditional β-catenin mutant mice (referred to as Lgr5CreER; Ctnnb1^Δ(ex3)^; YFP), where conditional gain-of-function β-catenin gene mutation resulted in the increased distribution of YFP-labeled cells in the liver (Figure 1D and 2B, Figure S3A-G), reflecting the importance of β-catenin signaling in regulating Lgr5+ hepatocyte proliferation. Immunostaining revealed that these YFP+ cells colocalized with Albumin-expressing hepatocytes (Figure 1E). Further, the colocalization of YFP fluorescent protein with phosphorylated β-catenin (p-β-catenin) demonstrated the effectiveness of our conditional gain-of-function mice (Figure 1E). To further validate the involvement of Wnt/β-catenin signaling in Lgr5+ hepatocyte proliferation, we mated Lgr5CreER; mTmG to Ctnnb1^flox/flox^ double-floxed mice to generate Lgr5CreER; mTmG; Ctnnb1^flox/flox^ mice (Figure S1). Tamoxifen injections led to the conditional deletion of floxed alleles in Lgr5-expressing cells, as indicated by our sections of the small intestine showing that the differentiation of Lgr5+ intestinal stem cells was blocked (Figure S4F). Immunostaining also demonstrated the effectiveness of β-catenin knockdown (Figure 2C). The distribution of Lgr5+ hepatocytes harboring a conditional inactivated allele of β-catenin almost disappeared in the liver (Figure 2A-B), suggesting that inactivation of Wnt/β-catenin signaling led to a failure in Lgr5+ hepatocyte expansion in the liver. Previous studies have shown that AKT/β-catenin signaling regulates liver resistance to ischemia/reperfusion injury 31. Considering the increased distribution of Lgr5+ hepatocytes in the damaged liver and the important role of Lgr5+ hepatocytes in repairing liver injury 25, we hypothesized whether AKT/β-catenin signaling pathway could regulate Lgr5+ hepatocyte proliferation to repair the liver injury. To test this possibility, we further explored whether AKT/β-catenin signaling plays an important role in regulating Lgr5+ hepatocyte proliferation. Immunostaining for phosphorylated β-catenin (p-β-catenin) antibody revealed PTEN-deficient hepatocytes with activated β-catenin signaling (Figure 2C). Further, we knocked out β-catenin and PTEN in Lgr5+ hepatocytes to simultaneously inhibit Wnt signaling and activate AKT signaling in order to determine whether β-catenin inhibition could reverse the increased proliferation of Lgr5+ hepatocytes induced by PTEN knockdown (Figure S1). The knockdown of β-catenin significantly reduced the effects of PTEN inhibition, and Lgr5+ hepatocytes were barely visible in the liver (Figure 2D, Figure S5). Quantification of labeled cells in the liver from Lgr5CreER; mTmG; PTEN^flox/flox^ and Lgr5CreER; mTmG; PTEN^flox/flox^; Ctnnb1^flox/flox^ mice demonstrated that β-catenin inhibition reversed Lgr5+ hepatocyte proliferation induced by PTEN knockdown (Figure 2E). Our genetic experiments employing β-catenin deletion in PTEN-deficient Lgr5+ hepatocytes revealed that PTEN-mediated AKT/β-catenin signaling endowed Lgr5+ hepatocytes with the capability to proliferate and expand.

## Discussion

**Figure 3:**
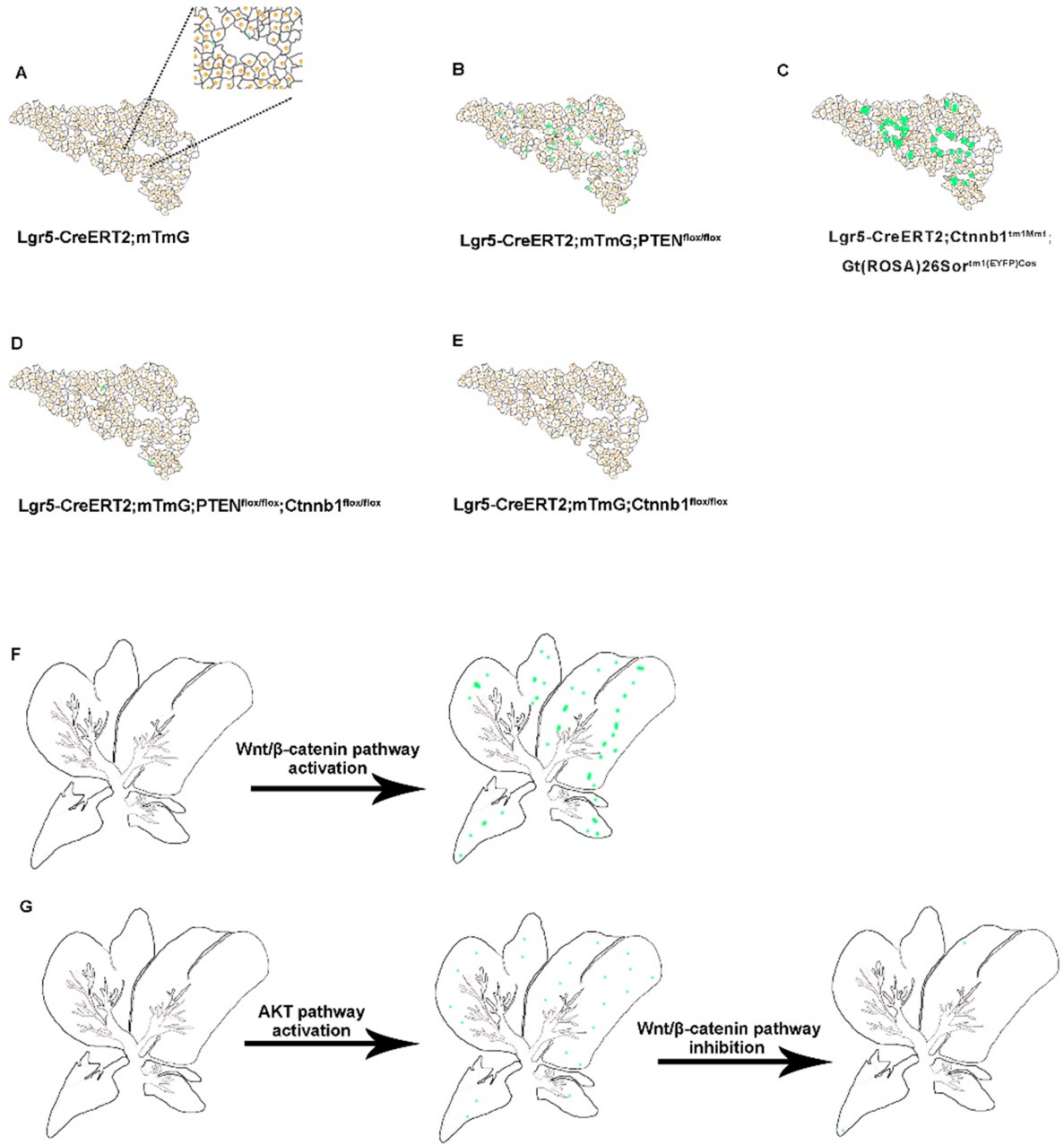
** Schematic summary of the distribution of Lgr5+ hepatocytes and their progeny under alteration of PI3K/AKT and Wnt/β-catenin signaling.** (A-E) Schematic depiction of the proliferative ability of Lgr5+ hepatocytes (green) during normal liver homeostasis (A) and under changes of PI3K/AKT and Wnt/β-catenin signaling (B-E). (F) Forced activation of β-catenin induced the expansion of Lgr5+ hepatocytes. (G) Schematic illustration of the crosstalk between Wnt/β-catenin and PI3K/AKT signaling pathways and their role in the proliferation of Lgr5+ hepatocytes.

Earlier Lgr5-IRES-creERT2 reporter mice suggested the expansion of the Lgr5+ cell population around the bile duct after liver injury, which can differentiate into relatively mature hepatocytes *in vitro* and *in vivo* 25. *In vitro*, Lgr5+ hepatocytes show stem cell potential, forming self-sustaining liver organoids from single-cell state, and differentiate into bile duct cells and hepatocytes after transplantation into FAH^-/-^ mouse model 25. Nevertheless, in the absence of liver injury, the proportion of Lgr5+ hepatocytes in the liver is enormously low, so this method has great limitations in practical application. Moreover, there are still many unanswered questions regarding the role of Lgr5+ hepatocytes in liver regeneration. Since the molecular mechanism of Lgr5+ hepatocyte activation after an injury is not clear, to recognize these signals and mechanisms has a significant meaning in the treatment of liver injury diseases. The expression pattern of Lgr5 after liver injury indicated that Lgr5 expression increased in the early stage after injury but decreased after tissue regeneration 25, 34. Therefore, certain signaling factors released by liver injury are necessary for the existence of Lgr5+ hepatocytes, and the specific signaling factors that function in liver regeneration need to be further explored. The outcomes of our study showed that the activated PI3K-AKT signaling after PTEN inhibition stimulated the proliferation of Lgr5+ hepatocytes (Figure 3A-B). At the same time, β-catenin overexpression also increased the distribution of Lgr5+ hepatocytes in the liver (Figure 3C and 3F). Therefore, Lgr5+ hepatocytes may undergo cellular proliferation based on the levels of AKT signaling and Wnt signaling in the damaged environment.

Studies suggest that the deletion of PTEN activates AKT-mediated β-catenin signaling in the ischemic liver and ameliorates hepatic ischemia/reperfusion injury, indicating that the AKT/β-catenin signaling pathway has a specific role in regulating liver inflammation 31. Our study found that the distribution of Lgr5+ hepatocytes after PTEN inhibition or β-catenin overexpression was akin to the expression pattern of Lgr5 in the liver after injury (Figure 3B-C and 3F), indicating that PI3K/AKT and Wnt/β-catenin signaling may be inflammatory signals released due to liver injury. Considering the effect of PTEN-mediated β-catenin signaling on innate immune responses in the damaged liver 31, we also found that the effects of PTEN deletion and β-catenin activation in Lgr5+ hepatocytes were synergetic (Figure 3D and 3G). Therefore, regulating PTEN-mediated AKT/β-catenin signaling can be considered a good way to control the proliferation of Lgr5+ hepatocytes for liver damage repair. Since Lgr5+ hepatocytes proliferate only briefly during injury 25, 34 and undergo less number cell division during homeostatic conditions 24, 26, the overactivation of AKT/β-catenin signaling is likely to disrupt the homeostasis of Lgr5+ hepatocytes with disastrous consequences, such as tissue proliferation in cancer. Recent studies have shown that Lgr5+ hepatocytes are highly sensitive to tumor transformation and are the main cells of origin in the development of hepatocellular carcinoma 24. Moreover, mouse hepatocellular carcinoma cells have similar expression patterns with damage induced Lgr5+ hepatocytes 35. Significantly, Lgr5 is among the highest upregulated genes in hepatocellular carcinoma associated with mutations in the Wnt/β-catenin pathway 36. PTEN deficiency also promotes the development of hepatocellular carcinoma 37, 38, suggesting that the abnormal proliferation of Lgr5+ hepatocytes triggered by AKT/β-catenin signaling may be a factor leading to liver cancer. This study may provide insights into how AKT/β-catenin signaling pathways drive the development of hepatocellular carcinoma.

Taken together, our data suggested that PTEN-mediated AKT/β-catenin signaling regulated Lgr5+ hepatocyte proliferation. Activation of PI3K/AKT and Wnt/β-catenin signaling led to the proliferation of Lgr5+ hepatocytes, which was very similar to the increased distribution of Lgr5+ hepatocytes after liver injury. Although the recent study reports a novel BAC transgenic model that is capable of tracking Lgr5+ hepatocytes in the liver 24, and our Lgr5 knock-in reporter mice were not able to faithfully exhibit the expression level of Lgr5 in the liver, that did not bother our awareness of the effects of AKT/β-catenin signaling on Lgr5+ hepatocyte proliferation. In the future, we may regulate the proliferation of Lgr5+ hepatocytes through AKT/β-catenin signaling in the damaged liver so as to promote damage repair. The study provides a theoretical basis for inducing Lgr5+ hepatocytes in the damaged liver to produce a positive proliferation response for tissue repair. Also, the prevention of excessive proliferation of Lgr5+ hepatocytes may provide a future treatment modality for liver cancer.

## Experimental procedures

### Mice

Mice were bred and maintained at the Shenzhen Center for Disease Control and Prevention in accordance with their guidelines. All mice were placed in SPF-class housing of the laboratory. Lgr5-GFP-Cre-ERT2 (Lgr5CreER) (stock no. 008875) and ROSA26-mTmG (R26-mTmG) (stock no. 007576) strains were obtained from the Jackson Laboratory. Ctnnb1^flox/flox^ mice were a gift from Dr. Zhenge Luo (Institute of Neuroscience, CAS). Ctnnb1^tm1Mmt^; Gt(ROSA)26Sor^tm1(EYFP)Cos^ mice were gifted by Dr. Fang Liang (South University of Science and Technology). PTEN^flox/flox^ mice were gifted from Dr. Hong Wu (University of California, Los Angeles). Lgr5CreER transgenic mice were crossed with R26-mTmG reporter mice to generate Lgr5CreER; mTmG mice. To inactivate β-catenin and PTEN activity in Lgr5+ cells, Lgr5CreER; mTmG mice were bred together with the floxed homozygous mice. To perform lineage tracing and simultaneously activate β-catenin activity in Lgr5+ hepatocytes, Lgr5CreER mice were mated to Ctnnb1^tm1Mmt^; Gt(ROSA)26Sor^tm1(EYFP)Cos^ mice. Mice of both genders were randomly selected for all lineage tracing experiments with equal gender ratios. All experiments were conducted using 8-week-old mice. All procedures were approved by the Animal Ethics Committee of Shenzhen Center for Disease Control and Prevention.

### Genotype identification of mice

Genotyping of mice was performed using the One-Step Mouse Genotyping Kit (Vazyme Biotech co., Ltd). After a 3 mm mouse tail was cut into pieces, freshly prepared 200 µL 1x lysis buffer was added to it. The tails were incubated in a 55 °C water bath for 20 min after vortex oscillation. Then, the samples were placed in a 95 °C water bath for 5 min to inactivate Proteinase K. After the vortex oscillation of the pyrolysis products, centrifugation was performed at 12,000 rpm for 5 min at 4 °C, and the supernatant was collected for PCR reaction. Refer to the Jackson Laboratory's website for detailed reaction conditions and primers (http://jaxmice.jax.org/strain/).

### *In vivo* Lineage tracing

Intraperitoneal injection of 167 mg/kg tamoxifen (Sigma, 16.7 mg/mL in corn oil) was performed every 24 h to activate the CreER recombinase in mice. To perform lineage tracing, 8-week-old Lgr5CreER; mTmG mice were given intraperitoneal injections with tamoxifen for five consecutive days, and liver samples were collected for observation 21 days post-injection.

### Immunofluorescence staining

Liver samples were fixed overnight with 4% PFA, washed with PBS for 8 h, and immersed in 30% sucrose for 8 h before embedding in OCT. The frozen liver was sectioned at 10 mm by a microtome (Leica). The slices were placed at room temperature for 30 min and then soaked in PBS for 10 min. Sections were blocked with block solution at 37 °C for 60 min and then incubated with primary antibody solution overnight. Primary antibodies used were: phospho-β-catenin (Cell Signaling, 5651, 1:100), phosphor-AKT (Cell Signaling, 4060, 1:200), Albumin (Proteintech, 16475-1-AP, 1:50). The sections were washed with PBS for three times, followed by incubation with secondary antibody (Jackon ImmunoResearch, 111-605-003, 1:200) at 37 °C for 60 min, and washed with PBS for three times. DAPI was added to the samples to incubate the cells in the dark for 15 min at room temperature. All images were acquired on a Zeiss confocal microscopy.

### Quantitation and statistical analysis

GraphPad Prism software was used for statistical analysis. Statistical significance was determined by unpaired Student's t-test. All results are expressed as means ± SEM, and n = 3 for each genotype. The proliferation rate of Lgr5+ hepatocytes was quantitated in whole-mount sections from the mice with different genotypes by counting mGFP- or YFP-labeled cells in the liver (three liver slices per biological replicate). The area of each section being counted is approximately 1,000 µm × 1,000 µm.

## Supplementary Material

Supplementary figures and tables.

## Abbreviations

PTEN: phosphatase and tensin homolog deleted on chromosome ten
Lgr5: leucine-rich repeat- containing G protein-coupled receptor 5
BAC: bacterial artificial chromosome
DDC: 3,5-diethoxycarbonyl-1,4-dihydrocollidine
FAH: fumarylacetoacetate hydrolase
GFP: green fluorescent protein
