# Supplementary material for: PTEN-mediated AKT/β-catenin signaling enhances the proliferation and expansion of Lgr5+ hepatocytes
Source: Int J Biol Sci. 2021 Feb 17;17(3):861–8. doi: 10.7150/ijbs.56091 (PMC7975694; doi:10.7150/ijbs.56091)
Supplement: Supplementary file 1 — Supplementary figures and tables. [file ijbsv17p0861s1.pdf]

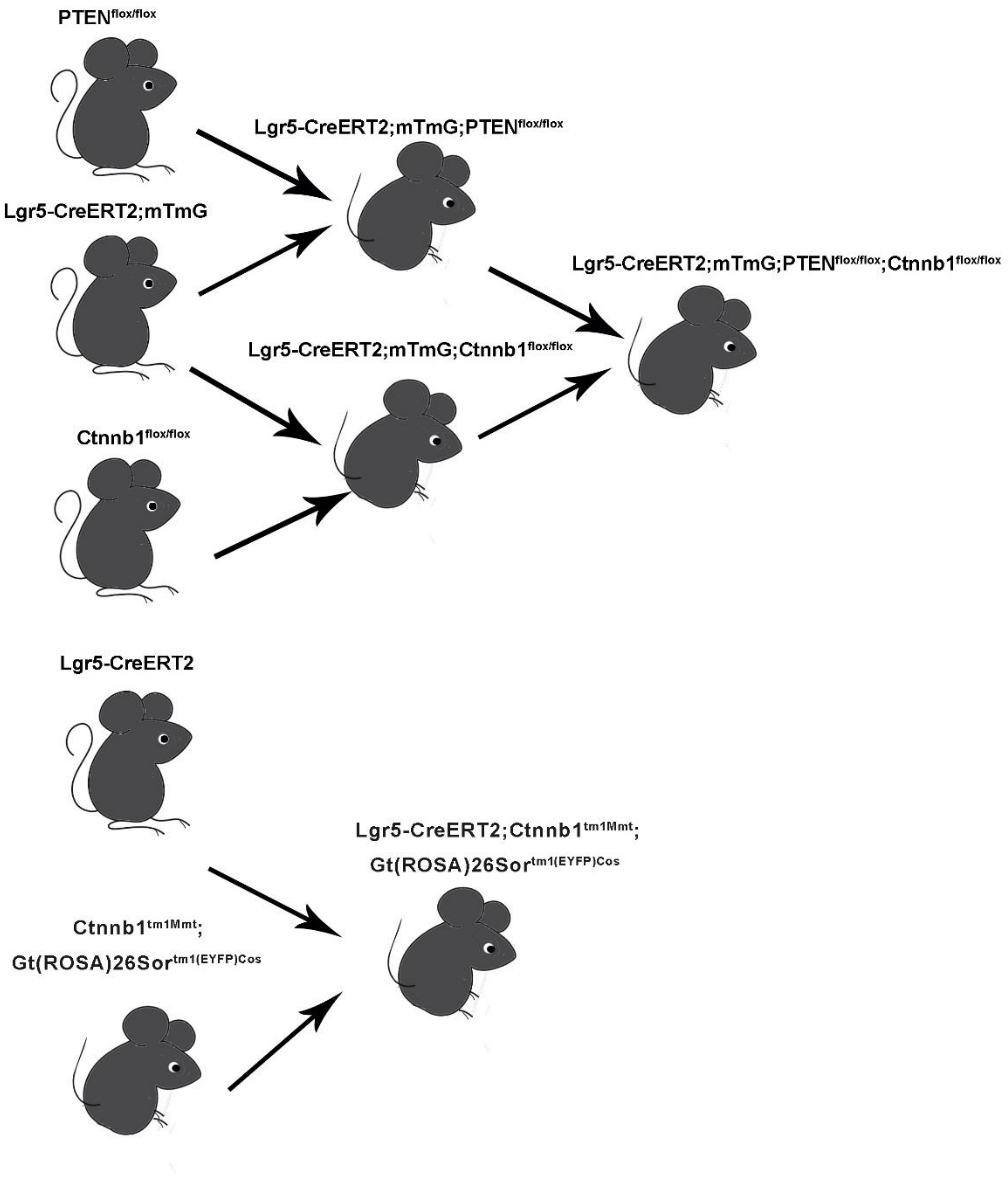

Supplementary Figure 1. Schematic diagram of the mouse hybridization in this experiment: Lgr5CreER; mTmG mice were crossed with PTEN $^{flox/flox}$ mice to obtain Lgr5CreER; mTmG; PTEN $^{flox/flox}$ mice. Lgr5CreER; mTmG mice were mated with Ctnnb1$^{flox/flox}$ mice to obtain Lgr5CreER; mTmG; Ctnnb1$^{flox/flox}$ mice. Then Lgr5CreER; mTmG; PTEN $^{flox/flox}$ mice were crossed with Lgr5CreER; mTmG; Ctnnb1$^{flox/flox}$ mice to obtain Lgr5CreER; mTmG; PTEN $^{flox/flox}$; Ctnnb1$^{flox/flox}$ mice. In order to overexpress an activated form of β-catenin in Lgr5-expressing cells, we crossed Lgr5CreER mice

with $Ctnnb1^{tm1Mmt}$; $Gt(ROSA)26Sor^{tm1(EYFP)Cos}$ mice to obtain Lgr5CreER; $Ctnnb1^{tm1Mmt}$; $Gt(ROSA)26Sor^{tm1(EYFP)Cos}$ mice.

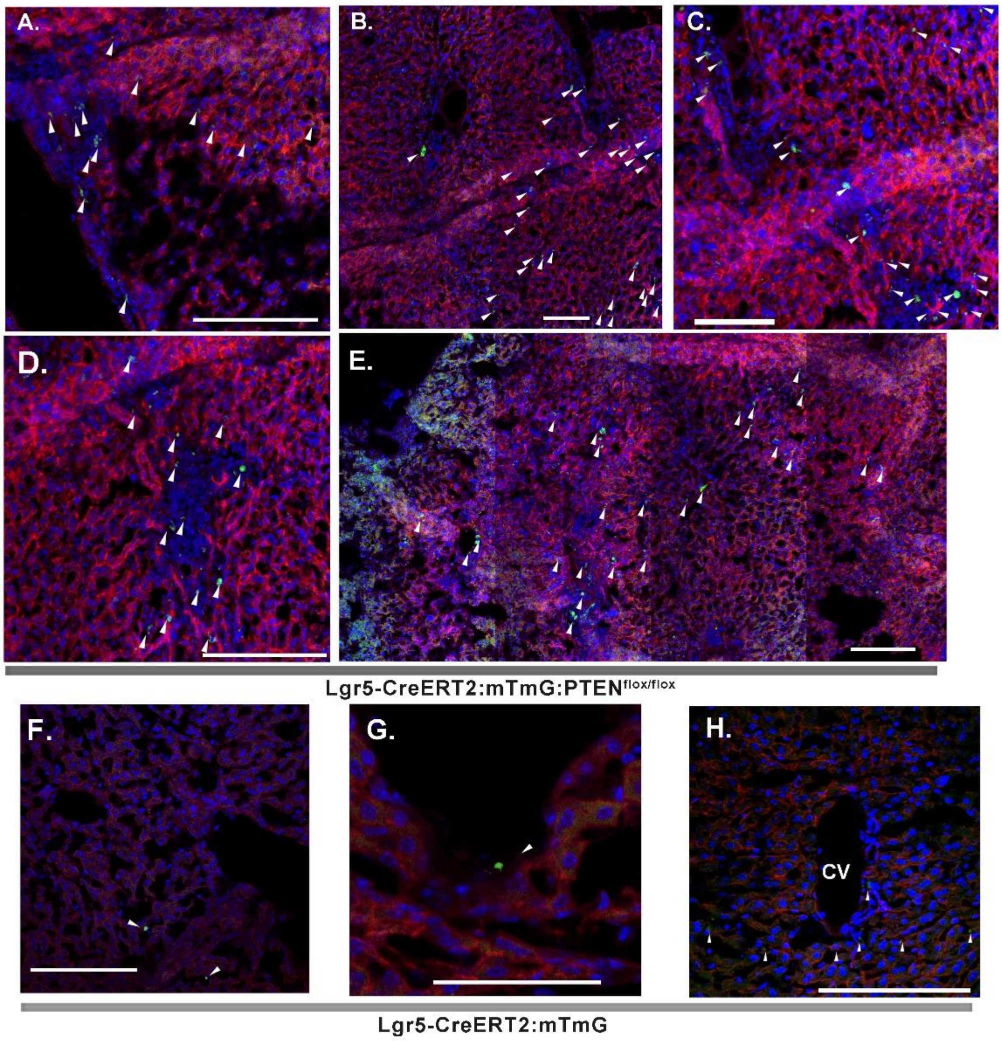

Supplementary Figure 2. Involvement of PTEN-mediated AKT signaling in Lgr5+ hepatocyte expansion. (A-E) Analysis of Lgr5+ hepatocyte fate in Lgr5CreER; mTmG; PTEN$^{flox/flox}$ reporter mice. Importantly, the number of mGFP+ cells increased compared to control Lgr5CreER; mTmG mice. (F-H) Lgr5+ hepatocytes and their progeny are distributed sporadically in Lgr5CreER; mTmG mice, accounting for only a small part of the total liver. Scale bar: 50 μm.

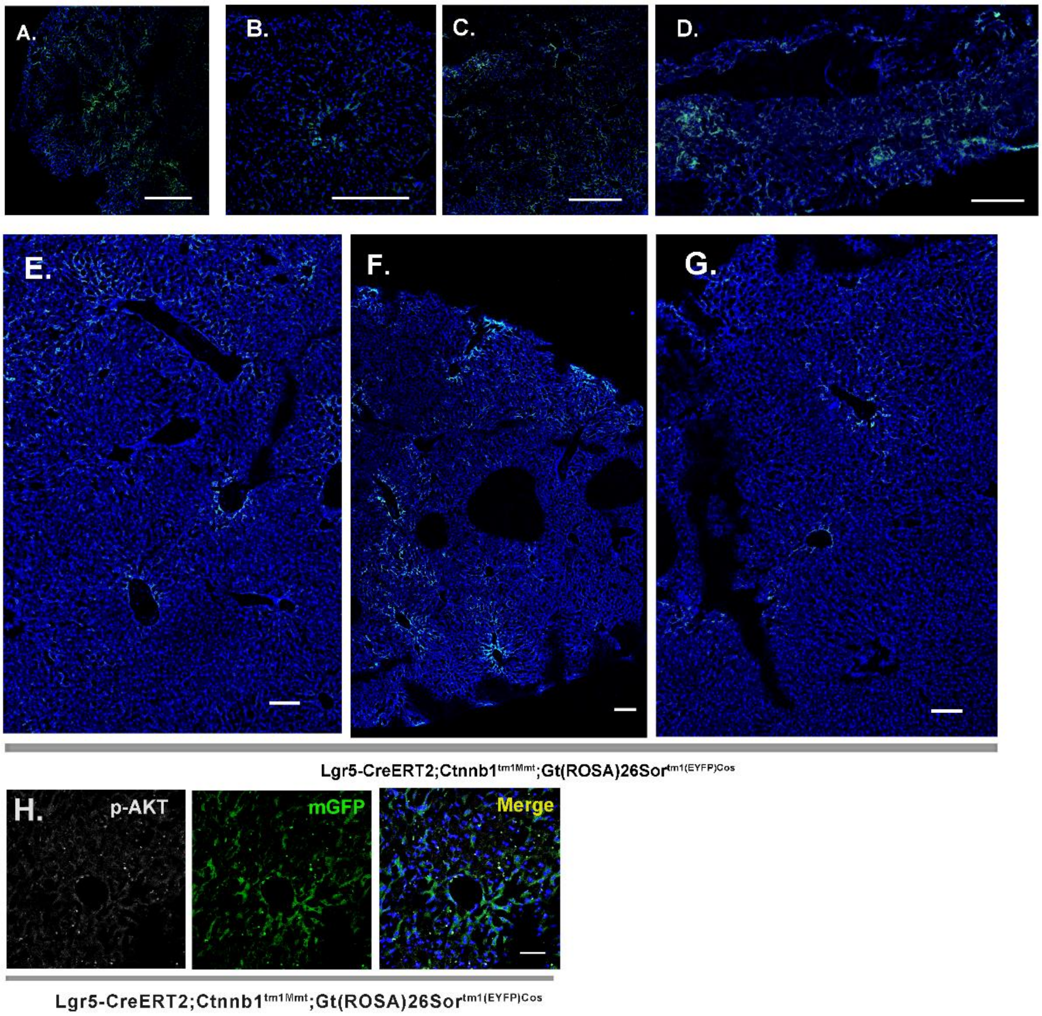

Supplementary Figure 3. Ectopic activation of β-catenin leads to expansion of Lgr5+ hepatocytes. (A-G) The increased distribution of Lgr5+ hepatocytes triggered by the activation of β-catenin. Scale bar: 50 μm.

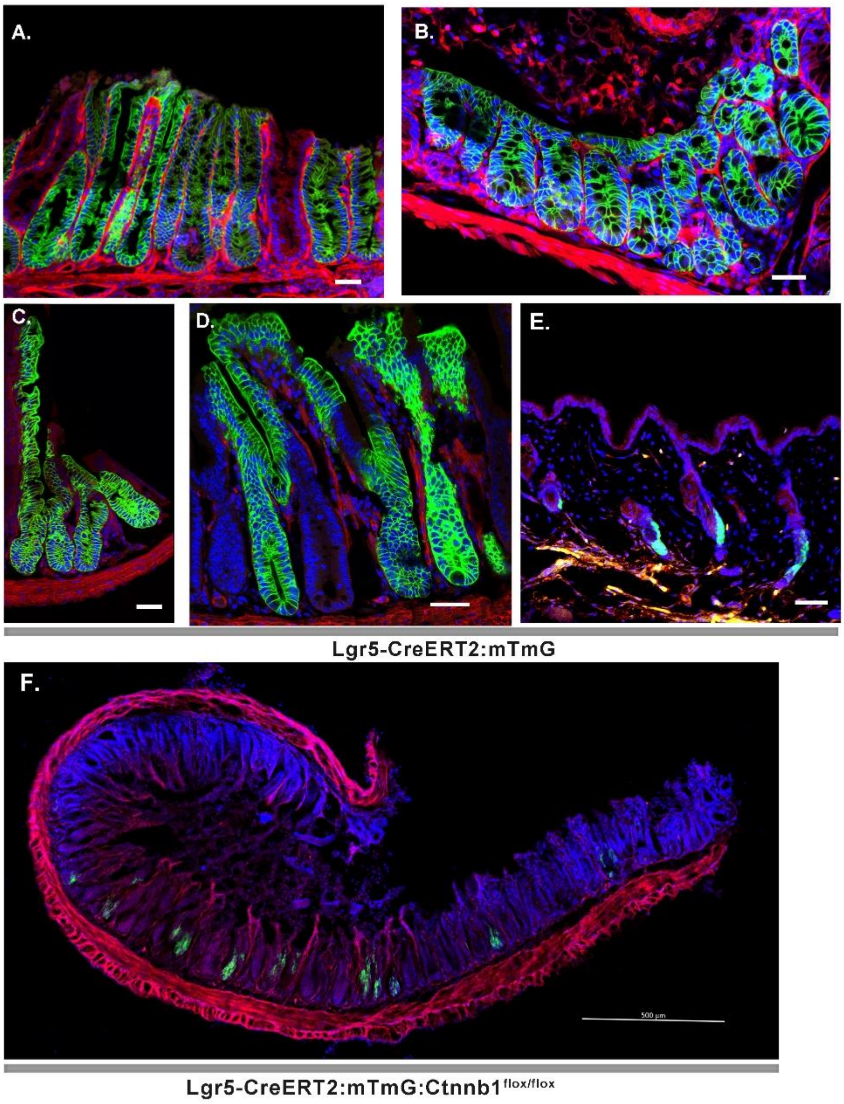

Supplementary Figure 4. Tracing of stem cells in the colon, small intestine, and hair follicle. Analysis of Lgr5-expressing stem cells and their progeny (green) in the (A-B) colon, (C-D) small intestine and (E) hair follicles. (F) β-catenin deletion in Lgr5+ stem cells significantly inhibited their differentiation in Lgr5CreER; mTmG; Ctnnb1$^{flox/flox}$ mice. Scale bar: 20 μm (A-E), 500 μm (F).

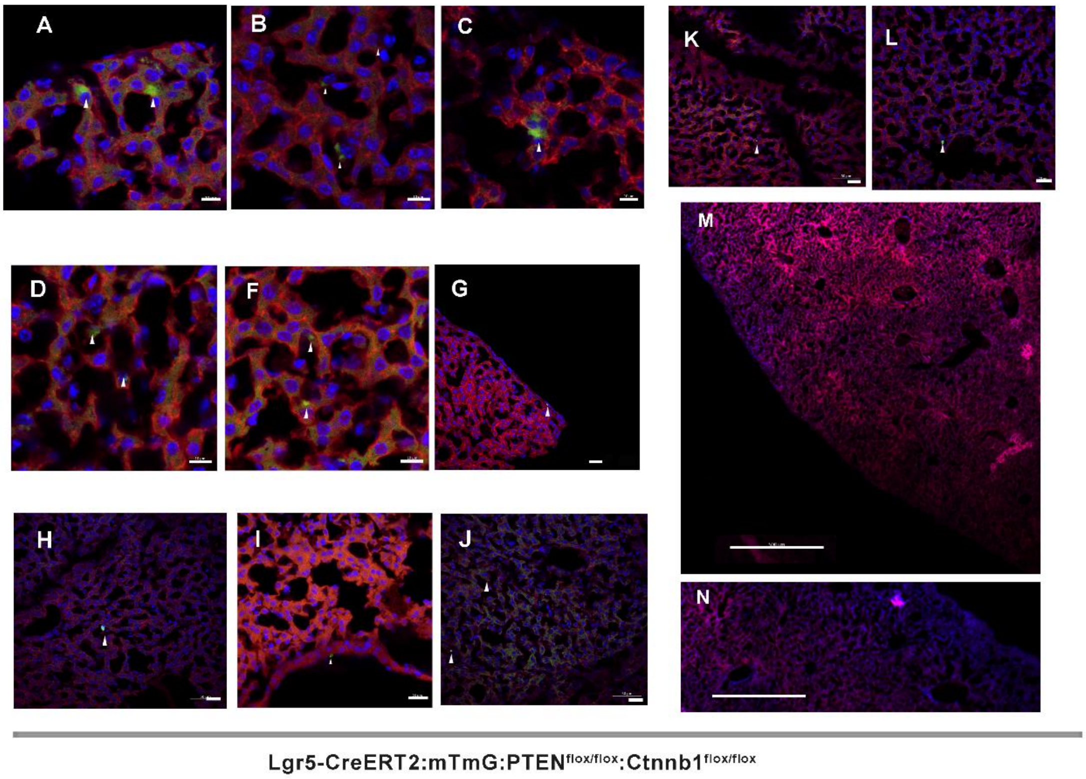

Supplementary Figure 5. The simultaneous inhibition of PTEN and β-catenin resulted in only sporadic Lgr5+ hepatocytes in the liver. Images of Lgr5-lineage cells in the liver upon PTEN and β-catenin deletion. Note that almost no mGFP-labeled cells were observed in the liver. Scale bar: 20 µm (A-L), 500 µm (M-N).
